# Synergy of Lepidopteran Nucleopolyhedroviruses AcMNPV and SpliNPV with Insecticides

**DOI:** 10.3390/insects11050316

**Published:** 2020-05-20

**Authors:** Beatriz Dáder, Eduardo Aguirre, Primitivo Caballero, Pilar Medina

**Affiliations:** 1 Unidad de Protección de Cultivos, Escuela Técnica Superior de Ingeniería Agronómica, Alimentaria y de Biosistemas, Universidad Politécnica de Madrid, 28040 Madrid, Spain; beatriz.dader@upm.es; 2 Institute for Multidisciplinary Research in Applied Biology, Universidad Pública de Navarra, 31006 Pamplona, Navarra, Spain; eduardo.aguirre@unavarra.es (E.A.); pcm92@unavarra.es (P.C.)

**Keywords:** baculovirus, *Autographa californica* multiple nucleopolyhedrovirus, *Spodoptera littoralis* nucleopolyhedrovirus, *Spodoptera exigua*, *Spodoptera littoralis*, Lepidoptera

## Abstract

The joint use of baculoviruses and synthetic insecticides for integrated pest management requires the study of the additive, synergistic or antagonistic effects among them on pest mortality. Droplet bioassays were conducted with *Autographa californica* multiple nucleopolyhedrovirus (AcMNPV), *Spodoptera littoralis* nucleopolyhedrovirus (SpliNPV) and seven insecticides (azadirachtin, *Bacillus thuringiensis*, cyantraniliprole, emamectin, metaflumizone, methoxyfenozide and spinetoram) on *Spodoptera exigua* and *Spodoptera littoralis*. The lethal concentrations LC_50_ and LC_95_ were calculated through probit regressions. Then, the sequential feeding of insecticides and nucleopolyhedroviruses was studied. Larvae were provided with the LC_50_ of one insecticide, followed by the LC_50_ of one nucleopolyhedrovirus 24 h later. The inverse order was also conducted. The insecticide LC_50_ and LC_95_ were higher for *S. littoralis* than for *S. exigua*. AcMNPV showed greater toxicity on *S. exigua* than SpliNPV on *S. littoralis*. Emamectin showed synergy with AcMNPV when the chemical was applied first, and metaflumizone and AcMNPV were synergistic regardless of the order of application, both from the first day of evaluation. SpliNPV was synergistic with azadirachtin and emamectin when it was applied first, but synergy was reached after 12–13 days. Excellent control is possible with the LC_50_ of azadirachtin, emamectin and metaflumizone in combination with nucleopolyhedroviruses, and merits further study as a means of controlling lepidopteran pests.

## 1. Introduction

The increasing use of synthetic organic chemicals has resulted in resistance and ecological concerns associated with environmental contamination and toxicity to non-target organisms [1]. Alternative control agents have been explored due to the growing demand for food free from chemical residues [1], a marked reduction in the number of active substances authorized for agricultural use and legislation promoting integrated pest management (IPM) as part of a framework of sustainable agricultural production [2].

The beet armyworm *Spodoptera exigua* (Hübner) and the cotton leafworm *Spodoptera littoralis* (Boisduval) (Lepidoptera: Noctuidae) are highly polyphagous key pests of many crops with economic importance in the Mediterranean basin such as sweet pepper, tomato or melon [3]. Chemical control measures in greenhouse horticultural crops in Spain resulted in resistance to the available insecticidal products, therefore entomopathogen-based insecticides such as *Bacillus thuringiensis* or baculoviruses have been commercialized [4,5,6].

Baculoviruses are double-stranded DNA viruses that control different orders of insects, including the larval stages of many lepidopteran pests of food crops. The family *Baculoviridae* comprises four genera, of which viruses of the *Alphabaculovirus* genus (lepidopteran nucleopolyhedroviruses, NPV) have shown considerable potential as bioinsecticides [7,8]. They are host-specific and have no adverse effects on natural enemies or other non-target insect populations, whereas the application of conventional insecticides reduces the abundance of beneficial agents [9,10].

Limitations to the use of baculoviruses include the cost of production, refrigerated storage and a relatively slow speed of kill [8]. One way to increase baculovirus insecticidal activity is the synergistic combination with low concentrations of synthetic insecticides. Although sublethal concentrations do not directly cause pest mortality, they may induce shifts in physiological and behavioral traits, compromising the pests’ fitness, and thus alter the course of pathophysiology during a subsequent viral infection [6,8,11,12,13,14,15,16,17,18]. Synergy is defined by the interaction of two or more pesticides to produce a combined mortality greater than the sum of their separate effects, which has been shown for azadirachtin and *Helicoverpa armigera* single nucleopolyhedrovirus (HearSNPV), *Spodoptera frugiperda* multiple nucleopolyhedrovirus (SfMNPV) and *Spodoptera litura* multiple nucleopolyhedrovirus (SpltMNPV) [11,12,13,14], the organophosphate chlorpyrifos and *Spodoptera litura* granulovirus (SpltGV) [15], and the spinosyn spinosad and *Spodoptera littoralis* nucleopolyhedrovirus (SpliNPV) and SfMNPV [16,17]. The mortality of the Guatemalan moth *Tecia solanivora* (Povolný) (Lepidoptera: Gelechiidae) was higher when granuloviruses isolated from *Phthorimaea operculella* (Zeller) (Lepidoptera: Gelechiidae) and *T. solanivora* were combined with the carbamate carbofuram or chlorpyrifos [18].

However, it is important that synthetic insecticides do not inactivate the viral pathogenicity of NPV when used together for IPM. Antagonism is defined by an interaction wherein two or more pesticides have an overall mortality that is less than the sum of their individual effects. For example, the carbamate methomyl was antagonistic when combined with *Autographa californica* multiple nucleopolyhedrovirus (AcMNPV) on *Heliothis virescens* (F.) (Lepidoptera: Noctuidae) [19]. *Anagrapha falcifera* multiple nucleopolyhedrovirus (AfMNPV) and *B. thuringiensis* were antagonistic against *Ostrinia nubilalis* (Hübner) (Lepidoptera: Crambidae), *Helicoverpa zea* (Boddie) (Lepidoptera: Noctuidae) and *Spodoptera frugiperda* (Walker) (Lepidoptera: Noctuidae) [20]. SpltMNPV was antagonistic with cartap hydrochloride on *Spodoptera litura* (F.) (Lepidoptera: Noctuidae) [15].

The literature on synergies has always considered the mixtures of insecticides provided to the pest at the same time. However, research on the sequential feeding of various compounds at different times is lacking. Besides, this situation more closely resembles real conditions, where farmers are likely to apply pesticides at different times throughout the crop cycle to avoid unexpected chemical reactions among active substances. Therefore, the goal of our study was to investigate the compatibility of two baculoviruses, AcMNPV and SpliNPV, with seven insecticides with different modes of action and widely used by producers in the Mediterranean basin, provided at different timeframes, to ascertain the synergistic, antagonistic or additive effects on the toxicity to second instar larvae of *S. exigua* and *S. littoralis*.

## 2. Materials and Methods

### 2.1. Insect Species

*Spodoptera exigua* was provided by Universidad Pública de Navarra (Spain) from a population collected in Almería (Spain). *Spodoptera littoralis* was collected on *Medicago sativa* L. in 2019 in Los Palacios (Sevilla, Spain). Both populations had not been previously exposed to insecticides and were continuously reared before the experiments at Universidad Politécnica de Madrid (Spain). Larvae were mass-reared in ventilated transparent plastic boxes (30 × 20 × 10 cm) on a semi-solid wheat germ-based semi-synthetic diet [21] inside walk-in chambers (4.25 × 2 × 2.5 m) at 25 ± 2 °C, 45 ± 1% relative humidity (RH) and 16L:8D photoperiod. After pupation, the emerged male and female adults were fed with a 50% honey solution inside ventilated methacrylate cages (40 × 30 × 30 cm). Filter paper was provided as oviposition substrate. For experiments, synchronized eggs laid on filter paper over 24 h were caged in plastic boxes. After hatching, the larvae were reared on the semi-synthetic diet under the same conditions as the general rearing.

### 2.2. Baculovirus Isolates

*Autographa californica* multiple nucleopolyhedrovirus (AcMNPV C6) and *Spodoptera littoralis* nucleopolyhedrovirus (SpliNPV) isolates were purified and occlusion body (OB) concentrations were determined at Universidad Pública de Navarra (Spain). Isolates were selected for being highly effective against *S. exigua* and *S. littoralis*, respectively, in previous bioassays. Suspensions of purified viruses were produced in fourth instar *S. exigua* and *S. littoralis* larvae, respectively. Virus-killed larvae were homogenized in distilled water, filtered through muslin and centrifuged in plastic vials at 3245 × *g* for 5 min with sodium dodecyl sulfate (0.1% w/v) to eliminate insect debris. The resulting pellets were washed in distilled water and re-suspended in Milli-Q water. OB concentrations were determined using an improved hemocytometer (Hawksley Ltd., Lancing, UK) under phase contrast microscopy and stored at 4 °C until the experiments were conducted.

### 2.3. Determination of the LC_50_ and LC_95_ of NPV and Insecticides at the Second Instar Larval Stage

Experiments were conducted at Universidad Politécnica de Madrid (Spain) from January to December 2019. AcMNPV, SpliNPV and seven insecticides with different modes of action were selected among the most frequently used for farmers to manage lepidopteran pests in the Mediterranean basin (Table 1). First instar larvae obtained from 24 h synchronized eggs were starved for 16 h. Newly molted second instar larvae were prompted out to drink 4 µL of distilled water droplets containing sucrose (15% w/v), blue food dye (0.001% w/v, ProGel^®^, Preston, UK) and a series of four to ten increasing concentrations of insecticides or NPV over 10 min, which caused between 5% and 100% mortality [22]. This droplet feeding method was selected to standardize a peroral intake procedure for all active substances tested. The second instar was selected for being the optimal larval stage for NPV efficacy. Previous bioassays were conducted to determine if the food dye was harmful to larvae (*n* = 56). Control larvae were treated identically but fed on a solution containing sucrose and food dye only, as the food dye was harmless. Larvae that ingested insecticide or mock water solutions turned blue because of the food dye. Only these larvae were individually transferred to blister packs and reared ad libitum on a semi-synthetic diet inside the walk-in chamber, as previously described at 25 ± 2 °C, 45 ± 1% RH and 16L:8D photoperiod. Larval mortality was checked three times a week until pupation (*n* = 56 larvae per concentration, using different batches of insects).

### 2.4. Combined Effect of Sequential Feeding of NPV and Insecticides

As previous work has always focused on the insecticide mixtures provided to the pest at the same time, we studied the sequential feeding of two compounds on different days to avoid the unexpected inactivation of active substances. Following the methodology described in Section 2.3, first instar larvae were starved for 16 h. Newly molted second instar larvae were prompted out to drink droplets containing the calculated 50% lethal concentration (LC_50_) of one of the insecticides. The larvae that ingested the solution turned blue and were reared on the semi-synthetic diet over 8 h, starved again for 16 h and then prompted out to drink droplets containing the calculated LC_50_ solution of one NPV (treatment 1: insecticide + NPV). The larvae that turned blue again were individually transferred to blister packs and reared ad libitum until pupation on a semi-synthetic diet inside chambers at 23.8 ± 0.1 °C, 54.8 ± 0.3% RH and 16L:8D photoperiod (model MLR-350, Sanyo Electric Co., Ltd., Osaka, Japan). The inverse order was also conducted, in which larvae were initially offered the LC_50_ solution of one NPV and prompted to ingest 24 h later the LC_50_ solution of one insecticide (treatment 2: NPV + insecticide), to determine the possible differences due to the speed of kill of the different compounds. Control treatments were solutions containing the LC_50_ of the single compounds (treatments 3 and 4: insecticide and NPV controls) and water (mock) (treatment 5). The experiment was conducted for every combination of insecticide/NPV. Larval mortality was checked three times a week until pupation (*n* = 84 larvae per treatment, using different batches of insects). The cause of death, by insecticide or NPV, was determined by the observation of symptoms (specific symptoms for each insecticide and, in the case of NPV, pale yellow/oily spots on the tegument, climbing to the upper lid of the blister pack to die or the complete disintegration/liquefaction of the larvae) and the presence of OBs inside cadavers under a microscope, because testing each larva for insecticide residue was not feasible with regard to the laboratory equipment and workforce. When larvae died showing the aforementioned symptoms and there was the presence of OBs, we assigned the cause of death to the NPV. When larvae died showing insecticide symptoms and there was no presence of OBs, we assigned the cause of death to the insecticide.

### 2.5. Statistical Analysis

Concentration mortality data from Section 2.3 were subjected to probit analysis using the POLO-Plus program (*p* ≤ 0.05) after assessing fit and overdispersion with other distributions such as logit, which did not provide a better fit than probit [24,25]. Pathogenicity expressed as the 50% and 95% lethal concentrations (LC_50_ and LC_95_), 95% fiducial limits and slopes of mortality curves of the insecticides and NPV were determined.

To determine the nature of the interactions between NPV and insecticides when assayed sequentially in Section 2.4, we tested the independent action model of two compounds by the comparison of the observed mortality with the expected probability of response of the combination [24]. The expected mortality was calculated by the equation:

*E* = [*O*_1_ + *O*_2_ (1 − *O*_1_)], (1)

where *O*_1_ and *O*_2_ are the observed mortalities after exposure to the single compounds. This model assumes that the probabilities of the effects of the two compounds are additive [19,26]. Biologically, this means that the insect dies when the amount of at least one compound exceeds the threshold of tolerance. Additive effects would predict an overall mortality of 75%, determined from the expected mortality of larvae treated with the two compounds. Significant deviations from that value would be indicative of antagonistic or synergistic effects.

The effects of the combinations were classified as antagonistic, additive or synergistic after obtaining χ^2^ values [27,28]. The difference between observed and expected mortalities was calculated by:

χ^2^ = (*O_mixture_* − *E*)^2/^*E*, (2)

where *O_mixture_* is the observed mortality of the combination and *E* is the expected response previously calculated. The tabular value of χ^2^ with *df* = 1 and *p* ≤ 0.05 is 3.84. The comparison of a pair of mortality values that resulted in χ^2^ < 3.84 would be indicative of additive effects, and χ^2^ > 3.84 would be indicative of significant synergy (*O_mixture_* − *E* > 0) or antagonism (*O_mixture_* − *E* < 0) (*p* ≤ 0.05).

## 3. Results

### 3.1. Determination of the LC_50_ and LC_95_ of NPV and Insecticides at Second Instar Larval Stage

Insecticides arranged from a higher to lower amount of the active ingredient needed to reach the LC_95_ on *S. exigua* were as follows, metaflumizone, cyantraniliprole, azadirachtin, *B. thuringiensis*, methoxyfenozide, emamectin and spinetoram (Table 2). The LC_95_ of four insecticides (azadirachtin, *B. thuringiensis*, cyantraniliprole and metaflumizone) and the LC_50_ of metaflumizone exceeded the maximum field concentrations according to national recommendations under our experimental conditions (Table 1 and Table 2).

The LC_50_ and LC_95_ of azadirachtin, *B. thuringiensis*, cyantraniliprole, methoxyfenozide and spinetoram were higher for *S. littoralis* than for *S. exigua* (Table 2 and Table 3). Again, azadirachtin, *B. thuringiensis*, cyantraniliprole, metaflumizone and methoxyfenozide exceeded the maximum field concentrations according to Spanish recommendations (Table 1 and Table 3). AcMNPV showed higher toxicity on *S. exigua* than SpliNPV on *S. littoralis* (Table 2 and Table 3). Preliminary bioassays showed that our AcMNPV isolate was not effective against *S. littoralis* as a much higher concentration was needed to kill 50% of the population (data not published).

### 3.2. Combined Effect of the Sequential Feeding of NPV and Insecticides

**Figure 1:**
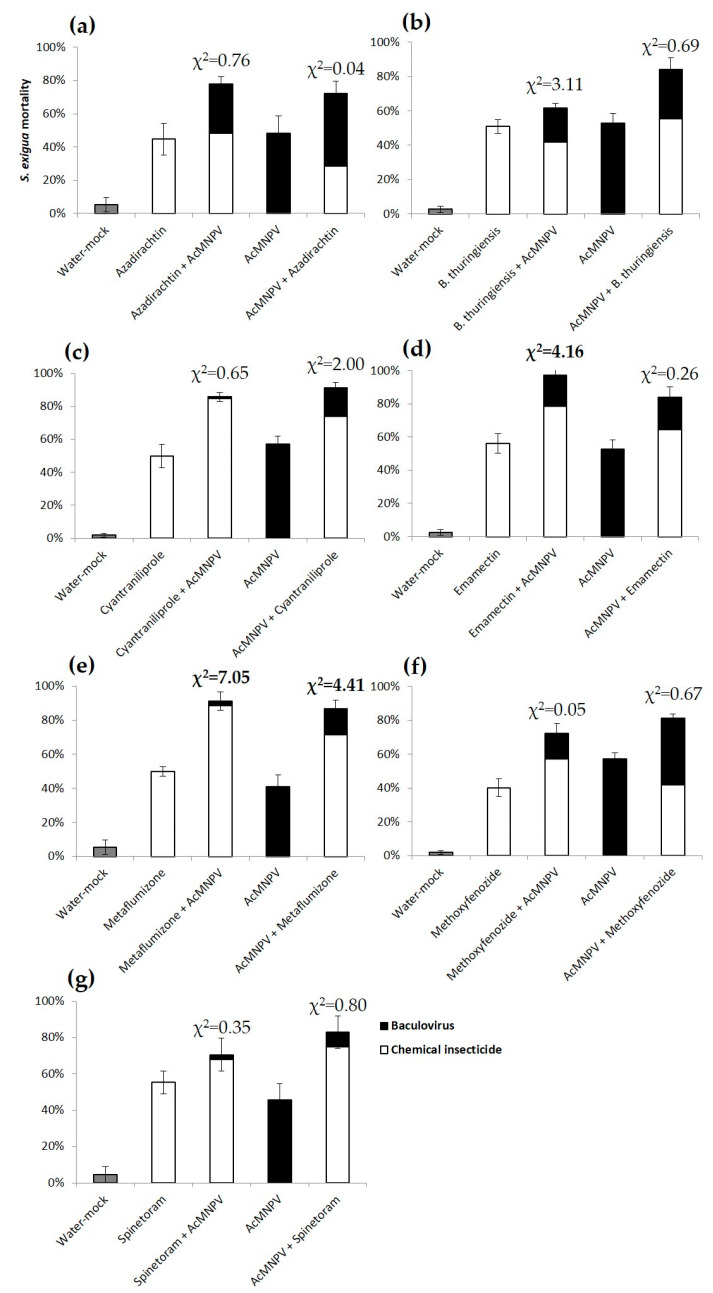
Mortality (%, mean ± SEM) of *Spodoptera exigua* second instar larvae provided with the LC_50_ of chemical insecticides, (**a**) azadirachtin; (**b**) *B. thuringiensis*; (**c**) cyantraniliprole; (**d**) emamectin; (**e**) metaflumizone; (**f**) methoxyfenozide; (**g**) spinetoram; the LC_50_ of AcMNPV or the sequential feeding of both over 10 min, and then maintained ad libitum on a semi-synthetic diet. The cause of death is represented by white bars for chemical insecticides and black bars for AcMNPV. Mock water controls are represented by gray bars. Significant χ^2^ showing synergy are in bold. Tabular χ^2^ with *df* = 1 and *p* ≤ 0.05 is 3.84.

**Figure 2:**
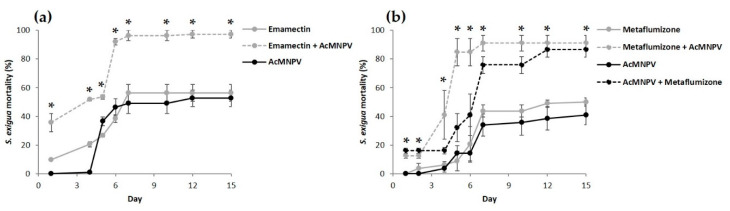
Cumulative daily mortality (%, mean ± SEM) of *Spodoptera exigua* second instar larvae provided with the LC_50_ of chemical insecticides (grey solid line), (**a**) emamectin; (**b**) metaflumizone; the LC_50_ of AcMNPV (black solid line) or the synergistic combination of both (gray or black dotted line) over 10 min, and then maintained ad libitum on a semi-synthetic diet. Asterisks stand for significant χ^2^, showing synergy.

The sequential feeding of AcMNPV with azadirachtin, *B. thuringiensis*, cyantraniliprole, methoxyfenozide or spinetoram resulted in the additive mortality of *S. exigua* and the differences between expected and observed mortalities were not significant, regardless of the order of application (Figure 1a–c,f,g). The sequential feeding of emamectin with AcMNPV showed significant synergy, only when the chemical was applied first (Figure 1d). Metaflumizone and AcMNPV also showed significant synergy, regardless of the order of application (Figure 1e). Emamectin and metaflumizone started killing larvae after 1–2 days, whereas the mortality due to AcMNPV started on day 4, reaching significant synergy from the first day of evaluation onwards (Figure 2a,b). In combined treatments, mortality due to AcMNPV was generally lower than that due to chemical insecticides, although the AcMNPV proportion was slightly higher in combination with azadirachtin, *B. thuringiensis* and methoxyfenozide compared to the rest of the chemicals (Figure 1a,b,f). The mortality of single treatments, either chemicals or AcMNPV applied alone, ranged between 40% ± 5% and 57% ± 4%. The water mock treatment mortality stayed below 5% ± 0% (Figure 1).

**Figure 3:**
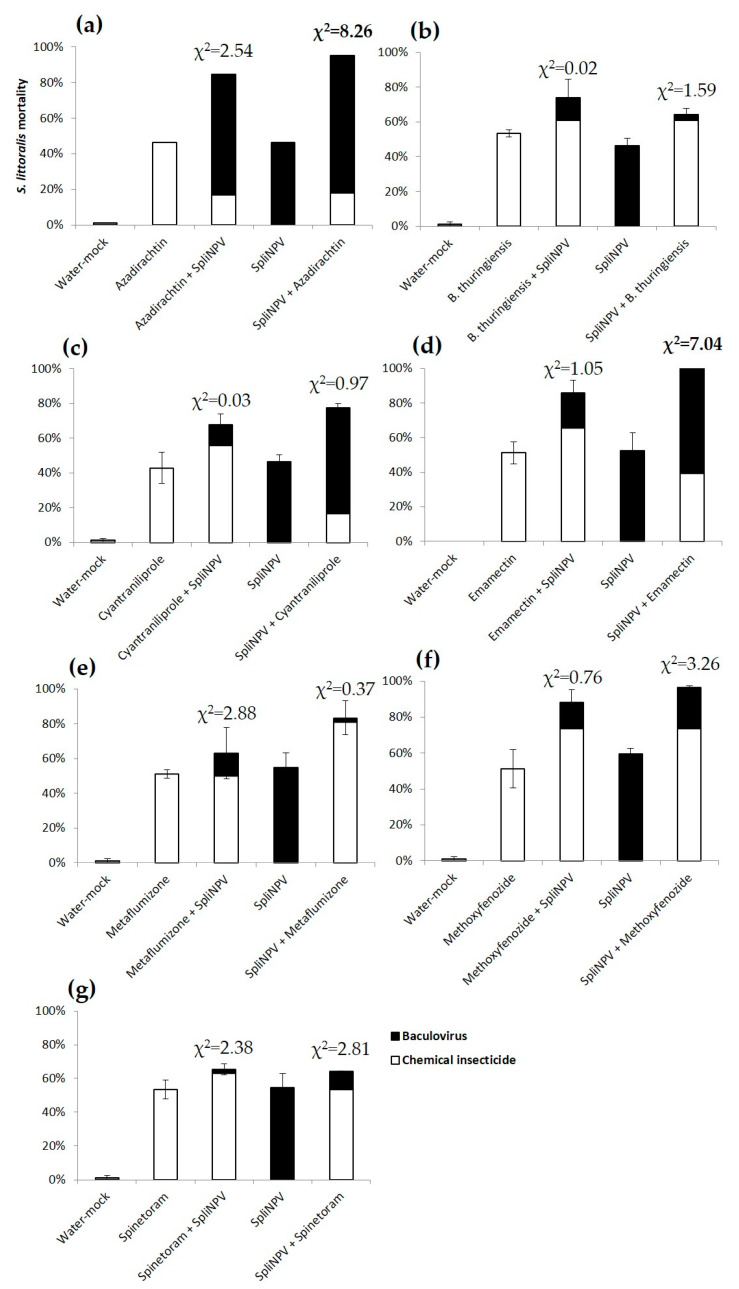
Mortality (%, mean ± SEM) of *Spodoptera littoralis* second instar larvae provided with the LC_50_ of chemical insecticides, (**a**) azadirachtin; (**b**) *B. thuringiensis*; (**c**) cyantraniliprole; (**d**) emamectin; (**e**) metaflumizone; (**f**) methoxyfenozide; (**g**) spinetoram; the LC_50_ of SpliNPV or the sequential feeding of both over 10 min, and then maintained ad libitum on a semi-synthetic diet. The cause of death is represented by white bars for chemical insecticides and black bars for SpliNPV. Mock water controls are represented by gray bars. Significant χ^2^, showing synergy, are in bold. Tabular χ^2^ with *df* = 1 and *p* ≤ 0.05 is 3.84.

**Figure 4:**
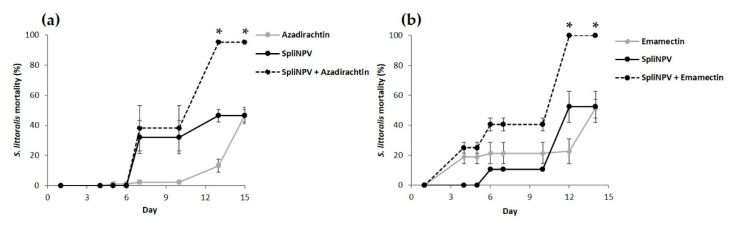
Cumulative daily mortality (%, mean ± SEM) of *Spodoptera littoralis* second instar larvae provided with the LC_50_ of chemical insecticides (gray solid line), (**a**) azadirachtin; (**b**) emamectin; the LC_50_ of SpliNPV (black solid line) or the synergistic combination of both (black dotted line) over 10 min, and then maintained ad libitum on a semi-synthetic diet. Asterisks stand for significant χ^2^, showing synergy.

For *S. littoralis*, we found a significant synergy between SpliNPV and azadirachtin, and SpliNPV and emamectin, both when the baculovirus was applied first (Figure 3a,d). Emamectin mortality started on day 4, azadirachtin on day 7 and SpliNPV after 6 days, which allowed synergy after 12–13 days (Figure 4a,b). The rest of the sequential combinations revealed additive effects (Figure 3b,c,e–g). The proportion of *S. littoralis* dead due to SpliNPV in the combined treatments was much more evident than in the case of AcMNPV on *S. exigua*, particularly for azadirachtin, cyantraniliprole and emamectin (Figure 3a,c,d). The mortality due to SpliNPV was lower than due to the chemical insecticides when it was combined with *B. thuringiensis*, metaflumizone and spinetoram (Figure 3b,e,g). The mortality of single treatments, either chemicals or SpliNPV applied alone, ranged between 43% ± 8% and 60% ± 3%. The mock water treatment mortality stayed below 1% ± 0% (Figure 3).

## 4. Discussion

Baculoviruses are valuable insect control agents for IPM, as part of a framework of sustainable agricultural production, due to their high specificity and overall safety for human and non-target organisms [2,8]. Their relatively slow activity can be overcome with a synergistic combination with low concentrations of synthetic insecticides [12,13,14,15,16,17]. In this work, toxicity bioassays were conducted with AcMNPV, SpliNPV and seven insecticides with different modes of action on the lepidopteran pests *S. exigua* and *S. littoralis*. Probit regressions were calculated prior to study the effect of the sequential feeding of NPV with these insecticides.

The LC_50_ of AcMNPV on *S. exigua* was approximately 10-fold lower than that of SpliNPV on *S. littoralis*. The lethal concentrations of insecticides were also higher for *S. littoralis* than for *S. exigua*. Emamectin and spinetoram were highly effective against *S. exigua* and *S. littoralis*. The LC_95_ was 5- and 9-fold below the national maximum field recommendations for emamectin, and 6- and 4-fold for spinetoram, respectively. On the contrary, the LC_95_ of azadirachtin, *B. thuringiensis*, cyantraniliprole, metaflumizone and methoxyfenozide greatly exceeded the recommendations. A 15-fold increase in metaflumizone and a 5-fold in cyantraniliprole was needed to reach the LC_95_ of *S. exigua*. For *S. littoralis*, larvae were treated with a 17-fold increase of azadirachtin and metaflumizone, and a 15-fold of methoxyfenozide. The differences can be explained due to the exposure method. In our droplet feeding method, larvae were exposed to insecticides for 10 min and the mortality was surveyed until the pupation of the survivors, whereas the toxicity studies on insecticides usually calculate acute mortality after 24–72 h and perform continuous exposure to the treated diet or leaves [12,15,16,29]. Thus, our larvae necessarily ingested less insecticide and a higher concentration was needed to calculate the LCs. Bioassays involving droplet feeding are well established for viral entomopathogens [20,22]. Moreover, the differences cannot be attributed to resistance, as our populations had never been exposed to chemical insecticides. Although explaining the higher susceptibility of *S. exigua* to NPV and insecticides is beyond the goal of this work, it should be taken into account that covert baculovirus infections in *S. exigua* in field and laboratory populations have frequently been described [30,31,32]. However, whether interactions in co-infected individuals influenced the insecticidal properties of the artificial infections conducted in our experiments, or latent infections reactivated from a covert state when the larvae were subjected to new infections, remains unknown [30,31,32].

The sequential feeding of NPV and insecticides was conducted to ascertain the additive, synergistic or antagonistic effects of binary combinations [27,28]. Synergism has different biological explanations: the same target site, physiological interactions, such as NPV infection, facilitating the absorption or distribution of the insecticide, the suppression of detoxification mechanisms or nerve cell infection resulting in an increased sensitivity to insecticide [19]. SpliNPV was synergistic with azadirachtin on *S. littoralis* when SpliNPV was applied first. Low levels of azadirachtin are synergistic with many NPVs. A decrease in the azadirachtin required when combined with gypsy moth NPV against *Lymantria dispar* (L.) (Lepidoptera: Erebidae) was observed [33]. Combining 0.1 ppm azadirachtin with 10^2^ OBs mL^−1^ SpltMNPV resulted in a 45% increase in the toxicity to *S. litura* [11,12]. The survival time of third instar *Helicoverpa armigera* (Hübner) (Lepidoptera: Noctuidae) was reduced by four days when 0.1 ppm azadirachtin was combined with 10^3^ PIBs mL^−1^ HearSNPV, compared to individual HearSNPV treatment [13]. A mixture of 1.1 mg L^−1^ azadirachtin and 177 OBs mm^−2^ SfMNPV showed synergy on third instar *Spodoptera frugiperda* (Smith) (Lepidoptera: Noctuidae) [14]. The literature reports lower concentrations of azadirachtin and NPV to reach synergy; but these authors offered treated leaves or a diet continuously compared to our single exposure of 10 min. Conversely, azadirachtin with AcMNPV did not increase the toxicity to *S. exigua* under our experimental conditions, similarly to *Heliothis zea* single nucleopolyhedrovirus (HzSNPV) on *Heliothis virescens* (F.) (Lepidoptera: Noctuidae) [29].

Emamectin showed synergy with AcMNPV on *S. exigua* when the chemical was applied first. The control of *S. littoralis* was also significantly improved with the sequential feeding of SpliNPV and emamectin. To our knowledge, this is the first evidence found concerning this insecticide, an activator of the glutamate-gated chloride channel [23]. Metaflumizone and AcMNPV were synergistic regardless of the order of application. The positive interaction of this semicarbazone, a voltage-dependent sodium channel blocker [23], with NPV had not been previously reported. Overall, the use of low concentrations of emamectin and metaflumizone in combination with NPV merits further study.

None of the combinations of NPV with insecticides, regardless of their mode of action, were antagonistic, suggesting that OBs were not inactivated, or viral pathogenicity was not negatively affected, by the chemicals when they were applied sequentially [18]. *Bacillus thuringiensis*, cyantraniliprole, methoxyfenozide and spinetoram had additive effects to NPV on larvae mortality. Literature on *B. thuringiensis* shows the opposite results; subspecies *aizawai* had synergy with *Spodoptera exigua* multiple nucleopolyhedroviruses (SeMNPV) and SfMNPV on *Spodoptera* larvae [34]. Subspecies *kurstaki* was synergistic to *Panolis flammea* nucleopolyhedrovirus (PaflNPV) on *Mamestra brassicae* (L.) (Lepidoptera: Noctuidae) [35]. Antagonism was found between the subspecies *kurstaki* and *aizawai*, and AfMNPV on *S. frugiperda* [20]. Besides, baculovirus insecticides expressing tailored *B. thuringiensis* Cry proteins have been developed with enhanced pathogenicity compared to the wild-type virus [36]. The literature on the rest of the compounds is scarce. There are no reports of synergism with spinetoram, a spinosyn that alters the function of nicotine and GABA-gated ion channels [23]. Doses of another spinosyn, spinosad, were reduced 3-fold when 10^3^ PIB mL^−1^ SpliNPV was added against *S. littoralis* [17]. Weak synergism was also detected in mixtures containing 3 ppm spinosad and 70 OBs mm^−2^ SfMNPV on *S. frugiperda* [16].

In general, the proportion of dead insects due to insecticides was higher than due to NPV in sequential treatments, probably because of the quicker action of synthetic insecticides [8,23]. Insects died from cyantraniliprole, emamectin, metaflumizone or spinetoram after 1–4 days of exposure, whereas AcMNPV-induced mortality started after 4–6 days and SpliNPV after 6–9 days. The quicker toxicity of emamectin and metaflumizone than NPV allowed synergy from the first day of evaluation onwards. In the case of emamectin, this was more pronounced for AcMNPV than for SpliNPV, because emamectin toxicity was slightly delayed in *S. littoralis*. On the other hand, we observed a slightly higher proportion of mortality due to NPV when they were combined with azadirachtin, *B. thuringiensis* or methoxyfenozide. The slower mode of action of these three compounds might have favored insect death due to viral infection [37]. The target protein responsible for the biological activity of azadirachtin is unknown [23]. We observed poor feeding and a concomitant lack of growth and molting under azadirachtin [37]. Azadirachtin prolonged the larval duration and this might have allowed the development of SpliNPV infection [13]. Indeed, azadirachtin and SpliNPV reached synergy after 13 days. *Bacillus thuringiensis* is a microbial disruptor of midgut membranes [23]. Even with the slower mode of action of *B. thuringiensis* compared to other synthetic insecticides, larvae usually died several days before the average time for NPV. Methoxyfenozide is an ecdysone receptor agonist involved in growth regulation [23]. Although it is known that NPV replication alters ecdysone-regulated host development [38], and one of the biological explanations of synergy is action on the same target site, we cannot conclude this might have caused an interaction with methoxyfenozide, based on the lack of current literature on synergies with this insecticide and our own results.

## 5. Conclusions

Overall, the synergy of low concentrations of azadirachtin, emamectin and metaflumizone with NPV can be an efficient means of controlling the lepidopteran pests *S. exigua* and *S. littoralis*. In our laboratory conditions, virtually complete control can be achieved with the LC_50_ of these compounds. The insecticide concentration needed to obtain synergy largely depends on the exposure method used, and the time to reach synergy relies upon the speed of death of the compounds. Verification in field conditions and the molecular interactions responsible for synergy remain to be examined, with the aim of ensuring their optimal incorporation in effective, safe and sustainable IPM programs.

## Figures and Tables

**Table 1:** Active ingredients, commercial products and mode of action according to IRAC classification of insecticides and nucleopolyhedroviruses tested against *Spodoptera exigua* and *Spodoptera littoralis* second instar larvae.

Active Ingredient	Commercial Product	Company	Mode of Action IRAC ^1^	MFRC ^2^
*Autographa californica* multiple nucleopolyhedrovirus (AcMNPV)	-	-	31	-
Azadirachtin 1% (azadirachtin A) [EC]	NeemAzal T/S^®^	Agrichem S.A. (Madrid, Spain)	Unknown	3 mL L^−1^
*Bacillus thuringiensis* subspecies *aizawai* (GC-91) 50% [WP]	Turex^®^	Mitsui Agriscience S.A. (Brussels, Belgium)	11A	2 g L^−1^
Cyantraniliprole 10% + Acibenzolar-S-methyl 1.25% [SC]	Minecto Alpha^®^	Syngenta S.A. (Madrid, Spain)	28	1 mL L^−1^
Emamectin 0.855% [SG]	Affirm^®^	Syngenta S.A. (Madrid, Spain)	6	1.5 g L^−1^
Metaflumizone 24% [SC]	Alverde^®^	BASF S.L. (Barcelona, Spain)	22B	1 mL L^−1^
Methoxyfenozide 24% [SC]	Runner^®^	Corteva Agroscience S.A. (Sevilla, Spain)	18	0.4 mL L^−1^
Spinetoram 25% [WP]	Delegate^®^	Corteva Agroscience S.A. (Sevilla, Spain)	5	0.4 g L^−1^
Spodoptera littoralis nucleopolyhedrovirus (SpliNPV)	-	-	31	-

^1^ Insecticide Resistance Action Committee [23], ^2^ Maximum field recommended concentration, according to Spanish authorities.

**Table 2:** Probit regressions of insecticides on *Spodoptera exigua* second instar larvae, showing the concentrations tested, the LC_50_ and LC_95_ with upper and lower fiducial limits at 95%, slope ± SEM, *t*-ratio and statistics according to POLO-Plus program (*p* ≤ 0.05).

Active Ingredient	Concentrations Tested	LC_50_ (95% Fiducial Limits)	LC_95_ (95% Fiducial Limits)	Slope ± SEM	*t*-Ratio	χ^2^	*df*	Heterogeneity
AcMNPV	1.7 × 10^3^, 1.7 × 10^5^, 1.7 × 10^7^, 1.7 × 10^9^ OBs mL^−1^	1.7 × 10^4^ OBs mL^−1^ (8.1 × 10^3–^3.4 × 10^4^)	1.6 × 10^6^ OBs mL^−1^ (4.3 × 10^5–^4.1 × 10^6^)	0.926 ± 0.122	7.572	0.292	2	0.146
Azadirachtin	0.375, 0.75, 1.5, 3, 6 mL L^−1^	2.108 mL L^−1^ (1.729–2.437)	4.635 mL L^−1^ (3.819–6.520)	4.807 ± 0.825	5.829	2.407	3	0.802
*Bacillus thuringiensis*	0.125, 0.25, 0.5, 1, 2, 4 g L^−1^	0.769 g L^−1^ (0.505–1.034)	3.616 g L^−1^ (2.395–8.058)	2.447 ± 0.320	7.644	4.307	4	1.076
Cyantraniliprole	0.1, 0.2, 0.4, 0.8, 1.6, 3.2, 6.4 mL L^−1^	0.384 mL L^−1^ (0.154–0.646)	4.964 mL L^−1^ (2.441–25.391)	1.479 ± 0.203	7.287	8.192	5	1.638
Emamectin	0.025, 0.05, 0.1, 0.2, 0.4 g L^−1^	0.082 g L^−1^ (0.069–0.096)	0.278 g L^−1^ (0.220–0.386)	3.109 ± 0.331	9.405	2.403	3	0.801
Metaflumizone	0.0625, 0.125, 0.25, 0.5, 1, 2, 4, 8, 16, 32 mL L^−1^	4.354 mL L^−1^ (1.982–6.274)	15.484 mL L^−1^ (10.237–46.487)	2.985 ± 0.538	5.551	14.08	8	1.760
Methoxyfenozide	0.01, 0.02, 0.04, 0.08, 0.16, 0.32, 0.64 mL L^−1^	0.077 mL L^−1^ (0.052–0.099)	0.398 mL L^−1^ (0.262–0.928)	2.300 ± 0.446	5.156	3.638	5	0.728
Spinetoram	0.005, 0.01, 0.02, 0.04, 0.08, 0.16 g L^−1^	0.008 g L^−1^ (0.006–0.011)	0.058 g L^−1^ (0.041–0.096)	1.928 ± 0.262	7.363	3.723	4	0.931

**Table 3:** Probit regressions of insecticides on *Spodoptera littoralis* second instar larvae, showing the concentrations tested, the LC_50_ and LC_95_ with upper and lower fiducial limits at 95%, slope ± SEM, *t*-ratio and statistics according to POLO-Plus program (*p* ≤ 0.05).

Active Ingredient	Concentrations Tested	LC_50_ (95% Fiducial Limits)	LC_95_ (95% Fiducial Limits)	Slope ± SEM	*t*-Ratio	χ^2^	*df*	Heterogeneity
Azadirachtin	0.75, 1.5, 3, 6, 12, 24, 48, 96 mL L^−1^	3.069 mL L^−1^ (2.240–4.064)	53.337 mL L^−1^ (33.593–101.411)	1.326 ± 0.129	10.31	4.010	6	0.668
*Bacillus thuringiensis*	0.125, 0.25, 0.5, 1, 2, 4, 8, 16 g L^−1^	2.604 g L^−1^ (1.348–3.581)	9.423 g L^−1^ (6.310–28.300)	2.945 ± 0.503	5.852	9.867	6	1.645
Cyantraniliprole	0.1, 0.2, 0.4, 0.8, 1.6, 3.2, 6.4 mL L^−1^	0.546 mL L^−1^ (0.410–0.699)	5.227 mL L^−1^ (3.535–9.199)	1.677 ± 0.182	9.192	3.495	5	0.699
Emamectin	0.003, 0.006, 0.012, 0.025, 0.05, 0.1, 0.2, 0.4 g L^−1^	0.054 g L^−1^ (0.038–0.068)	0.163 g L^−1^ (0.117–0.346)	3.430 ± 0.589	5.828	6.700	6	1.117
Metaflumizone	1, 2, 4, 8, 16, 32 mL L^−1^	3.155 mL L^−1^ (2.401–3.954)	17.453 mL L^−1^ (12.556–28.640)	2.214 ± 0.274	8.087	1.305	4	0.326
Methoxyfenozide	0.02, 0.04, 0.08, 0.16, 0.32, 0.64, 1.28, 2.56, 5.12, 10.24 mL L^−1^	0.858 mL L^−1^ (0.473–1.256)	6.199 mL L^−1^ (3.493–23.395)	1.915 ± 0.284	6.743	13.44	8	1.680
SpliNPV	1.0 × 10^2^, 1.0 × 10^3^, 1.0 × 10^4^, 1.0 × 10^5^, 1.0 × 10^6^, 1.0 × 10^7^ OBs mL^−1^	1.7 × 10^5^ OBs mL^−1^ (8.1 × 10^4–^3.2 × 10^5^)	7.0 × 10^6^ OBs mL^−1^ (2.8 × 10^6–^3.3 × 10^7^)	1.019 ± 0.102	9.977	4.719	4	1.180
Spinetoram	0.0025, 0.005, 0.01, 0.02, 0.04, 0.08, 0.16 g L^−1^	0.024 g L^−1^ (0.017–0.031)	0.098 g L^−1^ (0.068–0.184)	2.676 ± 0.292	9.170	6.467	5	1.293
